# DNA methylation regulator-based molecular subtyping and tumor microenvironment characterization in hepatocellular carcinoma

**DOI:** 10.3389/fimmu.2024.1333923

**Published:** 2024-04-26

**Authors:** Junsheng Zhao, Zhengtao Liu, Keda Yang, Sijia Shen, Jing Peng

**Affiliations:** ^1^ Key Laboratory of Artificial Organs and Computational Medicine in Zhejiang Province, Shulan International Medical College, Zhejiang Shuren University, Hangzhou, China; ^2^ State Key Laboratory for Diagnosis and Treatment of Infectious Diseases, National Clinical Research Center for Infectious Diseases, National Medical Center for Infectious Diseases, Collaborative Innovation Center for Diagnosis and Treatment of Infectious Diseases, The First Affiliated Hospital, Zhejiang University School of Medicine, Hangzhou, China; ^3^ Key Laboratory of Artificial Organs and Computational Medicine in Zhejiang Province, Shulan (Hangzhou) Hospital Affiliated to Shulan International Medical College, Zhejiang Shuren University, Hangzhou, China; ^4^ Department of Breast Surgery, Renji Hospital, School of Medicine, Shanghai Jiao Tong University, Shanghai, China

**Keywords:** hepatocellular carcinoma, DNA methylation modification, tumor microenvironment infiltration, immunotherapy, virus infection

## Abstract

**Backgroud:**

Although recent studies have reported the regulation of the immune response in hepatocellular carcinoma (HCC) through DNA methylation, the comprehensive impact methylation modifications on tumor microenvironment characteristics and immunotherapy efficacy has not been fully elucidated.

**Methods:**

In this research, we conducted a comprehensive assessment of the patterns of DNA methylation regulators and the profiles of the tumor microenvironment (TME) in HCC, focusing on 21 specific DNA methylation regulators. We subsequently developed a unique scoring system, a DNA methylation score (DMscore), to assess the individual DNA methylation modifications among the three distinct methylation patterns for differentially expressed genes (DEGs).

**Results:**

Three distinct methylation modification patterns were identified with distinct TME infiltration characteristics. We demonstrated that the DMscore could predict patient subtype, TME infiltration, and patient prognosis. A low DMscore, characterized by an elevated tumor mutation burden (TMB), hepatitis B virus (HBV)/hepatitis C virus (HCV) infection, and immune activation, indicates an inflamed tumor microenvironment phenotype with a 5-year survival rate of 7.8%. Moreover, a low DMscore appeared to increase the efficacy of immunotherapy in the anti-CTLA-4/PD-1/PD-L1 cohort.

**Conclusions:**

In brief, this research has enhanced our understanding of the correlation between modifications in DNA methylation patterns and the profile of the tumor microenvironment in individuals diagnosed with HCC. The DMscore may serve as an alternative biomarker for survival and efficacy of immunotherapy in patients with HCC.

## Background

DNA methylation is known to involve in multiple biological processes, including cancer progression (1), through the modification of chromosomal proteins, which alter the 3-dimensional conformation of the genome and/or protein-DNA interactions (2). DNA methylation is a type of dynamic reversible process in mammalian cells and is regulated by transmethylases, demethylases and recognizing proteins, which are defined as “writers”, “erasers” and “readers”, respectively (3). A recent study suggested that DNA methylation is a dynamic marker correlated with tumor immune escape and T-cell exhaustion (4).

Hepatocellular carcinoma (HCC) has a high incidence of malignant tumors and is the third leading cause of death among all types of cancers throughout the world (5). Approximately 80% of HCC cases are linked to persistent infections caused by either hepatitis B virus (HBV) or hepatitis C virus (HCV) (6). In addition, viral infection represents an important contributor to the epigenetic and tumor immune changes observed in HCC (7). Immune checkpoint inhibitors (ICIs) have been demonstrated to improve survival but only in a limited fraction of multiple tumor types, including HCC (8–10). There is growing evidence implying a link between DNA methylation and tumor immunity/immunotherapy. The innate immune response, which serves as a tumor suppressor, can be suppressed by the *DNA methyltransferase 1* (*DNMT1*), which maintains the silencing of retrotransposable elements (11, 12). In addition to PD-1/PD-L1 expression and tumor mutational burden (TMB), several methylation regulators, such as *ubiquitin-like with plant homeodomain* (*PHD*) and *ring finger domains 1* (*UHRF1*) and *Ten-eleven translocation* 1 *(TET1*), have been reported to be potential biomarkers for ICIs therapy (13, 14). Xu et al. found that the IFN-γ/JAK/STAT/TET2 signaling pathway is involved in influencing tumor immunity, and that stimulating *TET2* activity increases the efficacy of anti-PD-L1 drugs in solid tumors (15).

However, these previous studies have been limited by technical constraints and have focused only on a small number of methylation regulators and cell types. Nevertheless, because the antitumor effect of these agents involves multiple tumor suppressors working together in a well-coordinated manner. It is essential to gain a comprehensive understanding of how different DNA methylation regulators impact the tumor microenvironment (TME) to optimize the efficacy of immunotherapy for HCC. In this research, we utilized an unsupervised clustering technique to analyze gene expression data from 21 DNA methylation regulators in various cohorts to detect distinct patterns of modifications in DNA methylation regulators. We then evaluated the TME profiles represented by three distinct methylation regulator patterns. Finally, a DNA methylation score (DMscore) model was established to quantify the methylation status of the individuals. We validated the potential predictive value of the DMscore as an alternative biomarker for survival and immunotherapy efficacy.

## Materials and methods

### Data mining

The workflow of this study is shown in **Supplementary Figure 1A**. We analyzed 33 histologically confirmed HCC tissues and matched noncancerous liver tissue as normal controls. We named this cohort the “discovery cohort”, after which the preparation of mRNA libraries was performed followed by sequencing, and the gene expression profiles were analyzed as previously described (16, 17). The study received approval from the Research Ethics Committee at the First Affiliated Hospital of Zhejiang University, and written informed consent was acquired from all participating patients. Among the 33 patiens with HCC, 1 case was alcohol-induced cirrhosis and progressed to HCC, and the remaining 32 cases were HBV-related HCC. There were 27 males, compared with only six female patients. The lowest positive rate of eight serum markers was alkaline phosphatase (ALP), which was higher than the normal range in only two patients, and the positive rate was only 6% (2/33). The positive rate of alpha fetal protein (AFP) was the highest, which was 60% (20/33). All patients had grade A Child-Pugh scores. The clinical data of these patients were summarized in **Supplementary Table 7**.

Other gene expression data and full clinical annotations were downloaded from The Cancer Genome Atlas (TCGA), International Cancer Genome Consortium (ICGC) and Gene Expression Omnibus (GEO) databases. For the TCGA-LIHC (liver hepatocellular carcinoma) cohort, RNA sequencing (FPKM) of gene expression data were acquired using the R package TCGAbiolinks (18) and subsequently transformed into the more comparable transcripts per kilobase million (TPM) format. Somatic mutation and methylation 450K data were downloaded from the UCSE Xena browser. HBV/HCV infection was determined to positive if the participant of interest met the following criteria: HBV surface antigen; HBV DNA; HBV Core antibody; Hepatitis C Antibody; Hepatitis C Viral RNA; HCV Genotype (19). According to these criteria, 164 patients in the TCGA-LIHC cohort were HBV/HCV positive. For the HCC datasets in ICGC, RNA-seq data (raw counts), somatic mutation data and clinical information were obtained from the ICGC portal (https://dcc.icgc.org/projects/LIRI-JP). These specimens were predominantly obtained from a cohort of individuals in Japan who had been diagnosed with either HBV or HCV infection (20). The raw count format of the RNA-seq data was also transformed into TPM values. Batch effects between the TCGA-LIHC cohort and the LIRI-JP RNA-seq dataset were corrected using the “ComBat” function of the sva package (**Supplementary Figures 1B, C**). All the baseline information of the patients in the eligible HCC datasets is summarized in **Supplementary Table 1**.

### Tumor mutation burden analysis

To calculate the TMB of each patient, the total number of nonsynonymous mutations counted was divided by the exome size (38 Mb was utilized as the exome size). To calculate the TMB of each sample in the TCGA-LIHC cohort, we selected somatic mutation data processed by the VarScan platform and visualized them using the R package “maftools”.

The details of the mutations in the LIRI-JP cohort were visualized via a waterfall plot generated with the “GenVisR” package in R Studio software.

### GSVA analysis and functional annotation

To assess the level of activity in each biological pathway, the R package GSVA was used to compute the ssGSEA score for every sample (21). The gene set used to identify different immune cell types within the TME was obtained from Charoentong’s study and included 23 distinct subtypes of human immune cells such as activated CD8+ T cells, dendritic cells in an activated state, macrophages, natural killer T cells, and regulatory T cells, and so on (**Supplementary Table 2**) (22). The relative abundance of each immune cell type in TME was determined by an enrichment score acquired from ssGSEA analysis.

To investigate the disparities in biological mechanisms associated with DNA methylation, we conducted GSVA enrichment analysis employing the GSVA package. The “c2.cp.kegg.v7.4.symbols” gene sets were downloaded from the MSigDB database for GSVA.

### Unsupervised clustering of 21 DNA methylation regulators

Unsupervised clustering methods were used to identify different DNA methylation modification patterns and classify patients for further analysis. A set of 21 regulators (**Supplementary Table 3**) obtained from published literature was extracted from either the discovery cohort or the data-mining cohort to detect distinct DNA modification patterns facilitated by DNA methylation modifiers. We employed the ConsensuClusterPlus package to execute the aforementioned procedures, ensuring classification stability through 1000 repetitions (23).

### Identification of differentially expressed genes and enrichment analysis

We utilized the R package limma to detect gene expression related to DNA methylation modifications and identified pathways that were enriched with relevant associations. We also conducted KEGG analysis using the R package clusterProfiler, employing a significance threshold of *p* < 0.05 and FDR < 0.05.

### Generation of the DNA methylation score

Univariate Cox model analysis was used to assess the association between DEGs and overall survival. A total of 468 DEGs associated with a significant prognosis (*p*-value < 0.05) were identified for further analysis. Principal component analysis (PCA) was subsequently preformed to generate a signature relevant to DNA methylation. Both PC1 and PC2 were selected as signature scores. This approach offered the benefit of emphasizing the score on the subset containing a significant cluster of highly correlated (or anticorrelated) genes while reducing the impact of genes that do not exhibit similar patterns as other members within the subset. We defined the DMscore using a method similar to that reported previously (24–26):


$$\text{DMscore }=\;\sum^{}\;\left(PC1i+PC2i\right)$$

where ‘*i*’ represents the expression level of genes associated with methylation regulation and their impact on prognosis.

### Collection of genomic and clinical information on immune-checkpoint blockade

The present investigation employed five pretreatment tumor expression profiles obtained from cohorts receiving immune checkpoint blockade therapy to assess the agreement of the immunotherapy response. Data on anti-PD-L1 therapy efficacy in patients with metastatic urothelial tumors were obtained by the R package IMvigor210CoreBiologies (27). The sequencing raw count data were normalized and transformed into TPM values. We investigated the immunotherapeutic features of TCGA-LIHC patients by utilizing the publicly accessible Cancer Immunome Database (TCIA), which comprises relevant clinical pathology data.

### Statistical analysis

Statistical analyses were conducted using R software (version 4.0.3). The Wilcoxon test was used to compare differences between two groups. One-way ANOVA and Kruskal-Wallis tests of variance were used as parametric and nonparametric methods, respectively. Principal component analysis (PCA) was also conducted to investigate the distributions of the different groups using “prcomp”. Correlation coefficients between the TME-infiltrating immune cells and the expression of regulators were computed by Spearman and distance correlation analyses. We conducted both univariate and multivariate Cox regression analyses to identify potential risk factors. Additionally, we assessed the statistical significance of the differences insurvival rates across various risk groups using Kaplan-Meier (K-M) analysis with log-rank *p*-values. Venn diagrams, heatmapss, boxplots, forest plots, and alluvial diagrams were drawn using R. The immune cell profiles in TME of each sample in the data-mining cohort were analyzed using CIBERSORT (28). Next, MethylMix was utilized to analyze DNA methylation data and paired gene expression data to detect noteworthy DNA methylation occurrences that influence the expression of corresponding genes, thereby revealing their classification as genes driven by DNA methylation (29).

## Results

### Expression of DNA methylation regulators in the discovery cohort

**Figure 1:**
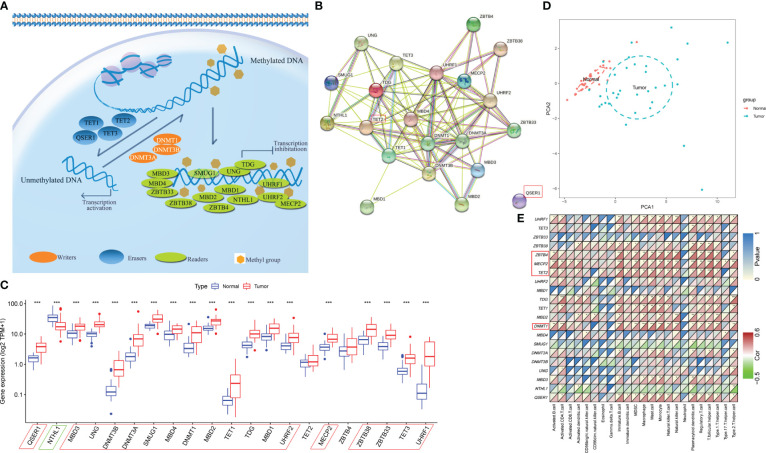
Landscape of DNA methylation regulators in HCC. **(A)** Summary of 21 DNA methylation regulators and their potential biological functions. **(B)** The PPI network downloaded from the STRING database indicates the interactions among the regulators. **(C)** The expression of 21 m6A regulators in normal and HCC tissues in the discovery cohort (*p-value < 0.05, **p-value < 0.01, ***p-value < 0.001). **(D)** Principal component analysis (PCA) of the expression profiles of 21 regulators to distinguish tumors from normal samples in the discovery cohort. **(E)** Spearman’s correlation heatmap between 21 DNA methylation regulators and immune cells in the discovery cohort (*p-value < 0.05, **p-value < 0.01, ***p-value < 0.001).

As shown in **Figure 1A**, 21 DNA methylation regulators were included in this study. As shown in **Figure 1B**, the protein-protein interaction (PPI) network revealed extensive protein interactions among methylation modifiers, with the exception of *QSER1*, which collaborates with *TET1* to inhibit *de novo* DNA methylation mediated by the enzyme *DNMT3* (30). Next, we performed a comparative analysis of the expression levels of DNA methylation regulators in paracancerous and HCC tissues within the discovery cohort (**Figure 1C**). Notably, HCC tissues presented significantly increased expression of *QSER1*, *MBD3*, *UNG*, *DNMT3B*, *DNMT3A*, *SMUG1*, *MBD4*, *DNMT1*, *MBD2*, *TET1*, *TDG*, *MBD1*, *UHRF2*, *MECP2*, *ZBTB38*, *ZBTB33*, *TET3* and *UHRF1*. Only *NTHL1* was expressed at significantly lower levels in HCC tumor tissue. Based on the expression levels of 21 DNA methylation regulators, we could distinguish HCC samples from normal liver samples (**Figure 1D**).

The ssGSEA algorithm was used to explore the association between the expression of these regulators and tumor immune cell infiltration in the TME. The expression of *MECP2*, *TET2*, *ZBTB4*, and *DNMT1* was positively correlated with more than half of the immune cells (**Figure 1E**). The immune cell profiles most strongly associated with these marks are immature B cells, MDSCs, Macrophages (Kupffer cells), Mast cells, T regulatory cells, Th17, and T follicular cells. These cell types are largely immunosuppressive and involved in immune evasion, and thus often associated with a poor prognosis in patients with HCC (31). Poorly correlations were found between most methylation regulators and key effectors of anticancer immunity include CD8+ T cells, Eosinophils, Nature killer cells (NK), and Dendritic cells (32–34).

### Landscape of DNA methylation regulators in data-mining cohort

**Figure 2:**
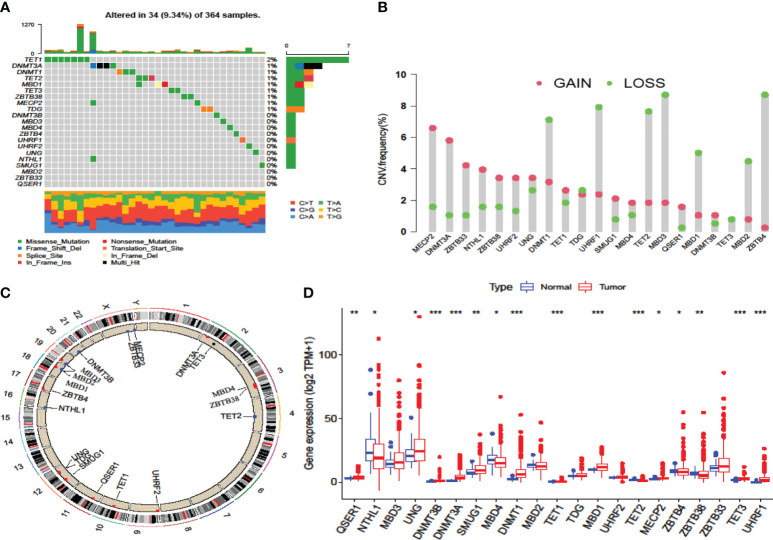
Multiomics landscape of DNA methylation regulators in the TCGA-LIHC cohort. **(A)** The mutation frequency of regulators in the TCGA-LIHC cohort. Each column represents the TMB. The number on the right shows the mutation frequency of each variant type. The lower bar represents the sample annotations. **(B)** The CNV frequency of the regulators in the TCGA-LIHC cohort. **(C)** The chromosomal locations of the regulators. **(D)** Differences in the gene expression levels of the regulators between normal and tumor patients in the TCGA-LIHC cohort. *p-value < 0.05, **p-value < 0.01, ***p-value < 0.001.

By examining the expression patterns of DNA methylation regulators and their correlation with TME cell infiltration in the discovery cohort, we proceeded to investigate the genetic modifications of these regulators in a data-mining cohort. Within the HCC genome of the TCGA-LIHC cohort, the overall mutation frequency among all the regulators was determined (**Figure 2A**). Among these regulators, the eraser *TET1* had the highest mutation rate (2%), and its mutation rate is a favorable prognostic marker of immunotherapy (14). Among the HCC patients, no mutations were observed in twelve other regulators, namely *DNMT3B*, *MBD3*, *MBD4*, *ZBTB4*, *UHRF1*, *UHRF2*, *UNG*, *NTHL1*, *SMUG1*, *MBD2*, *ZBTB33* and *QSER1* (**Figure 2A**). Analysis of copy number variation (CNV) alteration frequency revealed that regulators exhibited amplification in terms of copy number. Notably, *MDB1/2/3*, *TET2*, *UHRF1*, *DNMT1*, and *ZBTB4* exhibited widespread CNV deletion (**Figure 2B**). The chromosomal location of CNV among these regulators are shown in **Figure 2C**.

In the LIRI-JP cohort, the mutation rate of these regulators was similar to that in the TCGA-LIHC cohort (**Supplementary Figure 2A**). The reader *ZBTB38*, known as a tumor suppressor, had the most mutations, followed by *UHRF2*. However, no mutation in the eraser *TET1* was found in these patients. The chromosomal locations of these mutations are shown in **Supplementary Figure 2B**.

To ascertain whether the expression patterns of methylation regulators found in the discovery cohort also occurred in the data-mining cohort, we investigated the alterations in expression in the data-mining cohort. The results showed that most of these regulators were highly expressed in HCC tissues, while *MBD4* and *TET2* were expressed at significantly lower levels in HCC tissues (**Figure 2D**, **Supplementary Figure 2C**). As a prognostic risk factor and most mutated gene, *TET1* strongly inversely correlated with cytotoxic lymphocyte infiltration, which include CD8+ T cells and CD56dim NK cells, and positively correlated with Th2, which mostly related to tumor-promoting actives (35). In summary, these analyses revealed that variations in the genetics and expression of these DNA methylation regulators might play pivotal roles in the initiation, progression and heterogeneity of HCC.

### Methylation modification patterns mediated by 21 regulators

**Figure 3:**
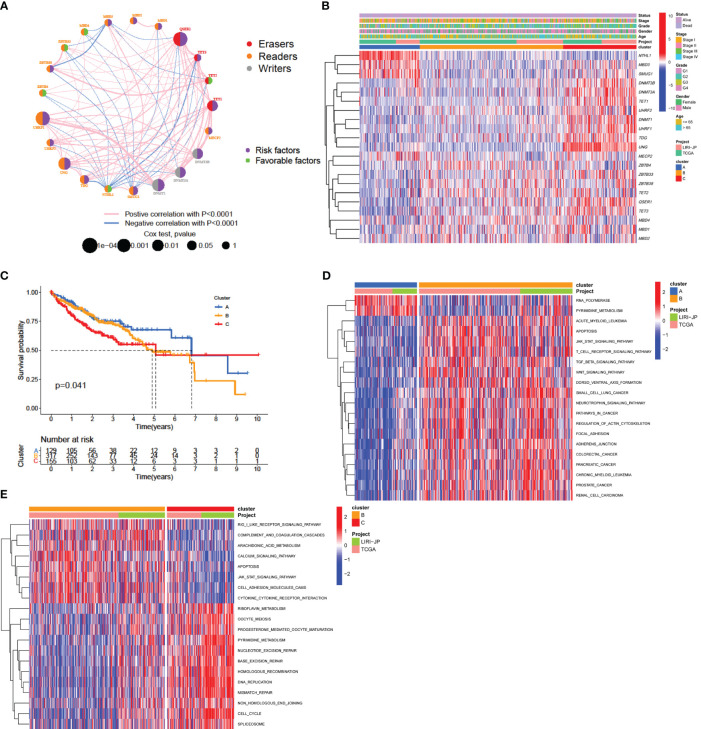
DNA methylation modification pattern and relevant biological characterization. **(A)** Correlations and prognosis of DNA methylation regulators in HCC patients. The red line represents a positive correlation with a *p*-value < 0.0001, and the blue line represents a negative correlation with a *p*-value < 0.0001. The size of the node represents the *p*-value value of the log-rank test. The right semicircle represents the prognostic factors for HCC: green represents favorable factors for OS, and purple represents risk factors for OS. **(B)** Consensus clustering of 21 regulators in 608 data-mining cohort samples. **(C)** Survival analysis of patients in the data-mining cohort according to distinct DNA methylation patterns. **(D, E)** GSVA enrichment analysis showing the activation states of biological pathways associated with distinct methylation modification patterns. A heatmap was generated to visualize these biological processes, in which red represents activated pathways and blue represented inhibited pathways. The data-mining cohort was used for sample annotation. **(D)** Cluster A vs. Cluster B; **(E)** Cluster B vs. Cluster C.

Gene expression and clinical information from 608 patients with HCC from the TCGA-LIHC and LIRI-JP cohorts were collected for analysis. Univariate Cox regression analysis revealed associations between the expression of 21 DNA methylation regulators and overall survival in patients with HCC (**Supplementary Figure 3A**). The results revealed that *TET1* and *QSER1* had the highest hazard ratios (HRs) of 1.431 and 1.209, respectively, while the absence of a regulator was found to be a significant favorable factor (*p*-value < 0.05). We created a network of methylation regulators to provide a comprehensive overview of the interactions and connections between DNA methylation and prognosis in patients with HCC (**Figure 3A**, **Supplementary Table 4**). We found that the expression of DNA methylation regulators was correlated not only with in the same functional category, but also among writers, erasers, and readers.

To explore additional insights into the potential patterns of DNA methylation regulation, an analysis was conducted using the expression profiles of the data-mining cohort. Three distinct expression patterns were divided using unsupervised clustering (**Supplementary Figures 3B-E**) and were termed Clusters A, B, and C, respectively (**Figure 3B**). Among these, 130 patients had pattern A, 320 had pattern B, and 158 had pattern C. An analysis of prognostic factors indicated a strong correlation between pattern A of DNA methylation regulators and intermediate survival duration (**Figure 3C**).

### Immune cell infiltration characteristics associated with distinct methylation modification patterns

We explored the differences in signaling pathways among the three patterns by GSVA enrichment analysis. As shown in **Figure 3D** and **Supplementary Table 5**, pattern A was enriched in nucleic acid biological processes, including RNA polymerase activity, ate biosynthesis, and pyrimidine metabolism. Pattern B was enriched for carcinogenic and immune fully activation, included the TGF-beta signaling pathway, focal adhesion and adheres junction, JAK-STAT signaling pathway, T cell receptor signaling pathway and cytokine-cytokine receptor interaction (**Figures 3D, E**). However, pattern C was strongly related to DNA replication and repair mechanisms, which included nucleotide excision repair, base excision repair, DNA replication, and mismatch repair (**Figure 3E**). The results of this research indicate that disruption of DNA methylation regulators can influence overall survival by affecting methylation levels, thus contributing to the significant variability observed in HCC.

**Figure 4:**
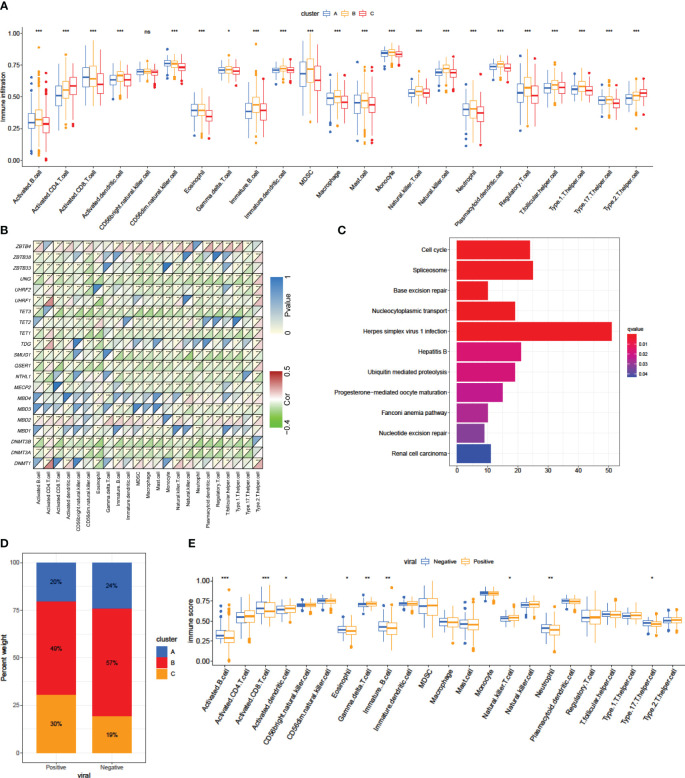
TME characteristics and transcriptome traits associated with DNA methylation modification patterns. **(A)** Boxplot of the relative immune cell abundances for the three DNA methylation patterns. (*p-value < 0.05, **p-value < 0.01, ***p-value < 0.001). **(B)** Spearman’s correlation heatmap between 21 DNA methylation regulators and immune cells in the data-mining cohort. Red indicates a positive correlation; blue indicates a negative correlation. (*p-value < 0.05, **p-value < 0.01, ***p-value < 0.001). **(C)** Functional annotation of DEGs among methylation clusters using KEGG enrichment analysis. The color depth of the bar plots represents the *q*-value of the enrichment. **(D)** The proportions of patients with the three modification patterns according to HBV/HCV viral infection status. **(E)** The difference in the number of infiltrating cells in each TME according to HBV/HCV viral infection status. The asterisks represent the statistical *p*-value: *p-value < 0.05, **p-value < 0.01, ***p-value < 0.001.

We utilized the ssGSEA algorithm to perform an extensive evaluation of the infiltration of TME cells. Consistent with favorable survival outcomes, cluster A exhibited the lowest level of activated CD4+ T cell infiltration and the highest level of activated CD8+ T cell infiltration (**Figure 4A**; **Supplementary Table 5**), could be classified as immune-inflamed phenotype. Surprisingly, TME cell infiltration analysis indicated that cluster B contained the most infiltrating immune cells, including dendritic cell, natural killer cell, eosinophils, B cell, MDSC, macrophage, mast cell, monocyte, neutrophil, Tfh (follicular helper T) cell, and so on (**Figure 4A**, **Supplementary Table 5**). However, patients in Pattern B did not show a matching survival advantage (**Figure 3C**). Previous research has indicated that tumors with an immune-excluded phenotype exhibit a significant abundance of immune cells in the stromal compartment encircling tumor cells (36). Therefore, cluster B might be identified as immune-excluded phenotype. Additionally, cluster C was similar immune-desert phenotype, distinguished by immunosuppression (**Figures 3D, E**, **Figure 4A**).

As DNA methylation regulators correlated with immune cell infiltration in the TME in the discovery cohort (**Figure 1E**), we further examined the correlation in the data-mining cohort. The expression of two writers, *DNMT3A* and *DNMT3B*, and three erasers, *TET1/3* and *QSER1*, was negatively correlated with most immune cells (**Figure 4B**). However, the expression of *ZBTB4*, *MBD2* and *DNMT1* was positively correlated with that of most immune cell types (**Figure 4B**), which was consistent with the findings in the discovery cohort.

A total of 1105 DEGs among the three DNA methylation patterns were identified, and KEGG pathway analysis revealed that those DEGs were enriched in HBV infection events (**Figure 4C**). The distinct etiological mechanisms of HCC, both viral and non-viral, exhibit a significant association with a unique TME (10). The level of immunosuppression observed in microenvironments associated with viruses was found to be considerably greater than that observed in unrelated microenvironments (37). Uninfected HCC exhibited a relatively reduced response to ICIs in comparison to infected patient with HCC (38). Further investigation revealed that patients with viral-infected HCC predominantly exhibited Pattern C methylation modifications, whereas those without viral infection were characterized by Pattern B modification patterns (**Figure 4D**). The expression of *DNMT1*, *DNMT3A*, *DNMT3B*, *MBD1*, *QSER1*, *TDG*, *TET1*, *TET2*, *TET3*, *UHRF1*, *UHRF2*, *UNG* and *ZBTB33* was significantly high in the virus-positive group (**Supplementary Figure 4A**).

The above results suggest that viral infection has the potential to impact the function of regulators responsible for DNA methylation, and might result in alterations in patterns of DNA methylation modifications. Additionally, we found the activated B cell, activated CD8+ T cell, eosinophil, immature B cell, neutrophil and Th17 were significantly down-infiltrated in viral-positive patients compared with viral-negative patients, while activated dendritic cell, gamma delta T cell and natural killer T cell was remarkably upregulated (**Figure 4E**). These results hold promise for advancing our understanding of the mechanisms underlying variations in methylation modification patterns observed in tumors.

After the completion of the aforementioned analyses, notable discrepancies were identified in the infiltration characteristics of TME cells with regard to patterns of DNA methylation modification. We subsequently employed the CIBERSORT technique to compare variations in immune cell components across the three patterns. The CIBERSORT analysis also showed significant correlation among DNA methylation regulators and immune infiltration (**Supplementary Figure 4B**). Conversely, macrophages M2 exhibited a significant enrichment of cluster A, while activated CD4+ memory T cells showed a notable enrichment of cluster B (**Supplementary Figure 4C**). Besides the stronger correlation to CD8+ T cell, and the differential expression of *QSER1* in virus infection status, we found the low expression of *QSER1* significantly prolonged OS (**Supplementary Figure 4D**). Taken together, these results indicated that DNA methylation modification did changes TME infiltration.

### Generation of DNA methylation gene signatures and functional annotation

As mentioned above, we identified 1105 DEGs associated with DNA methylation phenotypes using the limma package (**Supplementary Figure 5A**). The DEGs were related to the cell cycle, HBV infection, DNA replication and transcriptional events according to KEGG analysis (**Figure 4C**), revealing that the dissimilar clinical and transcriptomic features observed in the DNA methylation regulatory patterns could arise from variations in the distinct genes associated with the DNA methylation signatures. We identified 468 prognostic genes (*p*-value < 0.05) via univariate Cox model analysis (**Supplementary Table 6**). According to the expression levels of 468 genes, an unsupervised clustering analysis was conducted to categorize patients with HCC into three distinct clusters. These clusters, namely, DNA methylation geneCluster A/B/C, exhibit varying prognosis in terms of survival and immune-infiltrating features. (**Supplementary Figures 5B-J**). These findings provide additional support for the proposition that each HCC subtype exhibits distinct clinical and immune characteristics.

**Figure 5:**
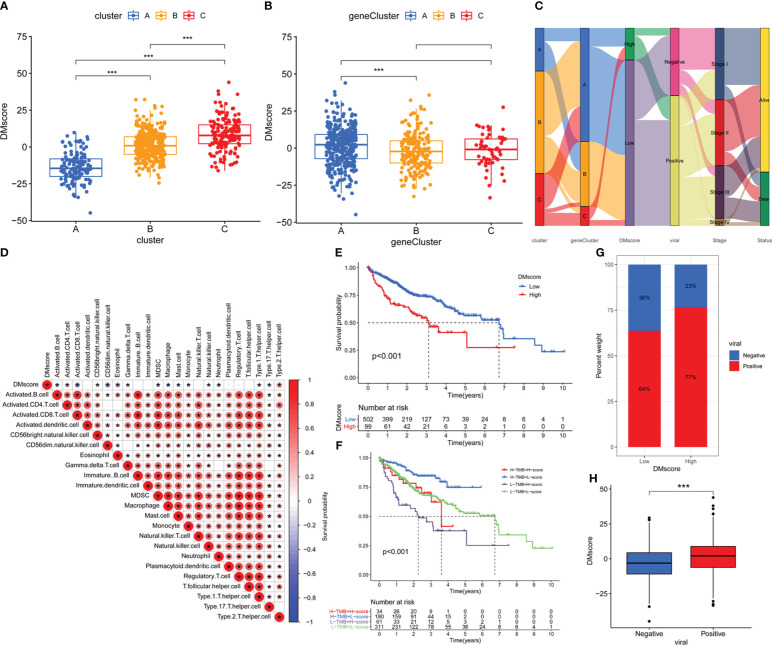
Construction of the DMscore. **(A)** Differences in the DMscore among the three methylation clusters. **(B)** Differences in DMscore among three geneClusters. **(C)** Alluvial diagram showing the changes in methylation clusters, geneCluster, DMscore, HBV/HCV viral infection status, disease stage and survival status. **(D)** Correlations between the DMscore and TME infiltration in the data-mining cohort. The asterisks represent the statistical *p*-value < 0.05 **(E)** Survival analysis of the high- and low-DMscore groups in the data-mining cohort. Log-rank test, *p*-value < 0.001. **(F)** Kaplan-Meier curves for patients in the data-mining cohort stratified by both TMB and DMscore. Log-rank test, *p*-value < 0.001. **(G)** Stacked bar plot depicting different proportions of patients with HBV/HCV infection in the high- and low-DMscore groups in the data-mining cohort. **(H)** Boxplot of the DMscores for distinct HBV/HCV infection status groups in the data-mining cohort, *p*-value = 2.7e-06. The asterisks in A, B, and H represent the statistical *p*-value: *p-value < 0.05, **p-value < 0.01, ***p-value < 0.001.

However, these studies were limited by their population size and lacked the ability to accurately predict the methylation modification patterns in individuals. To assess DNA methylation status at the individual level, we developed a risk score system called the DMscore, which is based on 468 signature genes associated with DNA methylation. Kruskal-Wallis test revealed significant discrepancy on DMscore between methylation regulator patterns. From this point of view, the median DMscore for DNA methylation regulator pattern C was found to be the highest, whereas methylation modification Pattern A exhibited the lowest median DMscore (**Figures 5A, C**). Gene cluster A showed the highest median DMscore (**Figures 5B, C**). An alluvial diagram was generated to illustrate alterations in attributes among individual patients (**Figure 5C**). Furthermore, we also tested the correlation between the TME ssGSEA score and the DMscore. As shown in **Figure 5D**, the DMscore was significantly correlated with the infiltration of most of the 23 immune cell types.

Subsequently, we assessed the prognostic value of the DMscore in patients with HCC. With the cutoff value of 10.87888 identified by the survminer package, HCC patients were separated into high and low DMscore groups. All the gene Cluster B samples belongs to the low DMscore group (**Figure 5C**). Patients in the low DMscore subgroup exhibited an extended duration of survival, with a 5-year survival rate that was twice as high as that in the high DMscore subgroup (7.8% compared to 3.0%) (**Figure 5E**). When TMB and DMscore were combined, the patients in the group with a low DMscore and high TMB exhibited superior overall survival compared to that of the remaining groups (**Figure 5F**, **Supplementary Figure 8**). These results suggested that the DMscore is a prognostic biomarker that could effectively predict survival of patient with HCC. In addition, we found that the DMscore was significantly correlated with viral infection status (**Figures 5G, H**).

Additionally, we analyzed TCGA-LIHC methylation data. Unsupervised clustering based on the methylation level of methylation-driven genes classified patients into two groups (**Supplementary Figure 6A**), which we called MethCluster C1 and C2. Similarly, the infiltration of most of the TME immune cells was significantly different between MethCluster C1 and C2 (**Supplementary Figure 6B**). In addition, MethCluster C1 had a significantly greater DMscore (**Supplementary Figure 6C**).

The present study demonstrated that the DMscore could reflect genomic methylation modifications, and effectively predict the survival of patients with HCC.

### Predictive value of the DMscore in immunotherapy

The individuals who will experience the most significant advantages from immunotherapy have been identified, as immnotherapy has improved clinical outcomes in the treatment of diverse tumor types. Considering the correlation between DMscore and immune infiltration, we further explored whether the DMscore could be a prognostic factor for immunotherapy efficacy by analyzing four ICI treatment cohorts.

**Figure 6:**
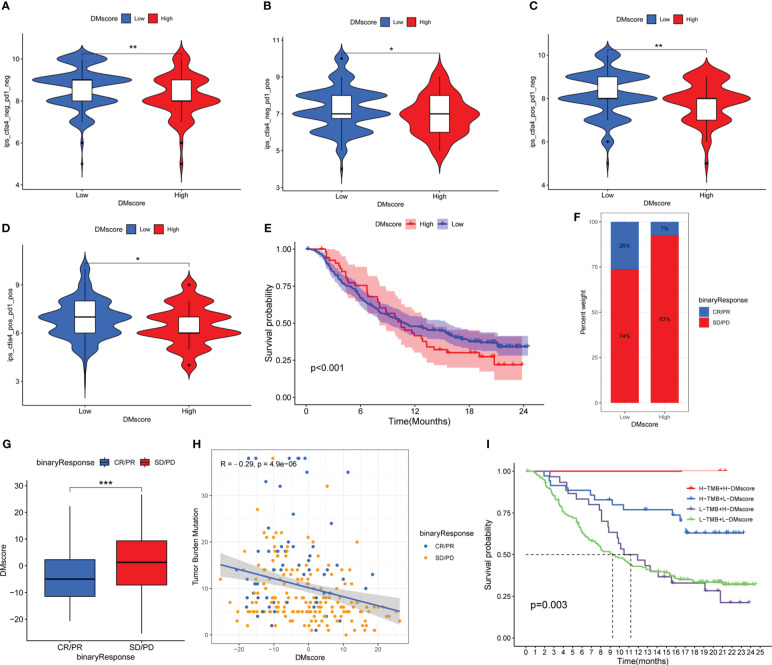
The effect of the DMscore in the immune checkpoint treatment cohorts. **(A-D)** Immunotherapeutic benefits of anti-CTLA-4/PD-1 therapy in the TCGA-LIHC cohort. **(E)** Survival analyses for patients with low- (32 patients) or high- (202 patients) DMscore. **(E)** Patient groups in the anti-PD-L1 immunotherapy cohort were evaluated via Kaplan-Meier curves (IMvigor210 cohort). Log-rank test, *p*-value < 0.001. **(F)** The proportion of patients who responded to PD-L1 antibody immunotherapy in the low- or high-DMscore groups. SD, stable disease; PD, progressive disease; CR, complete response; PR, partial response. **(G)** Distribution of the DMscore in distinct anti-PD-L1 clinical response groups. Wilcoxon test, *p*-value = 0.00067. **(H)** Correlations between the DMscore, TMB, and anti-PD-L1 immunotherapy response in the IMvigor210 cohort. **(I)** Survival analyses of patients receiving anti-PD-L1 immunotherapy stratified by both the DMscore and TMB using Kaplan-Meier curves. Log-rank test, *p*-value = 0.003. *p-value < 0.05, **p-value < 0.01, ***p-value < 0.001.

In the TCIA-LIHC cohort (anti-CTLA-4/PD-1 therapy), patients in the DMscore-Low group exhibited a significantly higher response rate (**Figures 6A–D**), indicating the immunotherapeutic benefits of CTLA-4/PD-1 antibody therapy in patients with a low DMscore.

Consistent with the above findings, a similar result was also obtained in the IMvigor210 cohort (anti-PD-L1 therapy). According to the K-M survival analysis of the anti-PD-L1 cohort (IMvigor210), patients with a low DMscore exhibited significantly improved overall survival (p-value < 0.001) (**Figure 6E**) and significant immunotherapeutic benefit (**Figures 6E-G**). Additional investigations into the measurement of TMB, a biomarker for immunotherapy efficacy, demonstrated a significant association between a decreased DMscore and increased TMB (**Supplementary Figure 7A**). A significant negative correlation was found between the DMscore and TMB (**Figure 6H**). Survival analysis revealed that patients with a combination of a high DMscore and high TMB had a great survival advantage (**Figure 6I**).

The detection of immune phenotypes in the IMvigor210 cohort was feasible because we could explore the variation in the DMscore across different phenotypes. Our results suggest that there is a notable association between decreased DMscore and immune phenotypes prevalent in desert regions, which may hinder the effectiveness of checkpoint inhibitors in combating tumors within this specific phenotype (**Supplementary Figure 7B**).

In the studies suggested a notable association between DNA methylation alterations and tumor immune characteristics as well as the efficacy of ICI immunotherapy. The utilization of the DMscore could aid in predicting the response to ICI immunotherapy and potentially enhance its predictive accuracy when used in combination with the TMB.

## Discussion

Ongoing research has consistently confirmed the significant role of abnormal DNA methylation in promoting genome instability and its impact on regulating antitumor immune responses and immunotherapy outcomes (14, 39, 40). However, there is still a need for a comprehensive understanding of the overall modulation of DNA methylation modification and the immune microenvironment among patients with HCC.

The initial phase of the research involved identifying 21 enzymes responsible for regulating DNA methylation that exhibited differential expression in both HCC carcinomas and paracancerous tissues. Subsequently, we validated the changes in methylation regulatory enzyme levels during HCC development by analyzing the expression of these enzymes in two publicly available datasets (TCGA-LIHC and LIRI-JP). In contrast to Sun et al.’s findings of *TET1* underexpression in HCC (41), our study consistently demonstrated overexpression of *TET1* in tumor tissues across all three datasets. Although the differential expression profiles of some genes were not entirely consistent across the three datasets and some genes showed differential expression in opposite directions, the 21 DNA methylation regulatory enzymes overwhelmingly showed significant differences in expression levels between cancer and paracancerous tissues in all three datasets. The variation in the orientation of disparities in expression could be attributed to the considerable diversity observed in HCC (42). The distinct expression patterns of these regulatory enzymes in HCC and adjacent noncancerous tissues suggest the significant involvement of DNA methylation regulatory enzymes in the progression of HCC.

In this research, we classified three unique patterns of DNA methylation modification by analyzing the expression levels of 21 regulators involved in DNA 5-mc methylation. Cluster A exhibit activated adaptive immunity, consistent with the immune-inflamed phenotype; Cluster B featured activation of innate immunity and stroma, corresponding to the immune-excluded phenotype; and Cluster C had immunosuppressive characteristics, similar to the immune-desert phenotype. The immune-excluded and immune-desert types could be classified as noninflamed tumor, namely, “cold tumors”. A “hot tumor”, characterized by significant infiltration of immune cells in the TME, exhibits an immune-inflamed phenotype (36, 43, 44). Consistently, patients with pattern A had the longest overall survival. Although pattern B exhibited significantly abundant immune cells, the activated TGF-β pathway resulted in reduced infiltration of T cells into tumors and weakened their ability to eliminate tumor cells. This leads to an immune-excluded phenotype and a less favorable prognosis (27, 45). The presence of immune-desert phenotypes featured with immune tolerance and ignorance, due to a lack of activated and primed T cells. Therefore, it is not surprising that patients in Cluster C had poorer survival outcomes than patients in other clusters (46).

In addition, it has been demonstrated that the variations in the mRNA transcriptome among the three DNA modification patterns are significantly linked to biological pathways associated with DNA replication and viral immunity. Similarly, similar to the clustering outcomes of the phenotypes resulting from DNA methylation modifications, we identified three genomic subtypes based on the expression levels of the DEGs in the mRNA. These subtypes also exhibit distinct survival prognoses and are significantly correlated with immune status. This once again highlighted the crucial role of 5-mc methylation modification in influencing diverse immune environments.

Considering the heterogeneity of methylation modification between individuals, we established a DNA methylation scoring (DMscore) system to calculate the methylation modification features of individuals with HCC. As expected, Cluster A was associated with geneCluster A/B and was characterized by immune activation and a lower DMscore, which corresponded to longer survival time (**Figures 3C**, **4A**, **5A-E**). The objective response rates (ORRs) observed in the CheckMate 040 trial indicated that patients treated with nivolumab, a PD-1 inhibitor, exhibited lower efficacy in patients infected with HBV/HCV than in those without viral infections (47). Consistent with these findings, we found that patients with HBV/HCV infection had relatively high DMscores (**Figures 5G-H**), while patients who benefited from anti-CTLA-4/PD-1 therapy tended to have relatively low DMscores (**Figures 6A-G**). The presence of a virus-induced immune-suppressing tumor microenvironment may explain the limited effectiveness of immunotherapy in treating HCC. These findings also reinforce the significance of using the DMscore as a predictive tool in guiding immunotherapeutic approaches. Moreover, when combined with the TMB, a well-established biomarker for assessing response to immunotherapy (48), the DMscore demonstrated enhanced accuracy in predicting both patient survival (**Figure 5F**)and treatment outcomes (**Figure 6I**). Another interesting result is that a low-DMscore intent to be higher response rate to atezolizumab but presented a “desert” immunophenotype in IMvigor210 cohort. One possible explanation for this result is that DMscore was negatively correlated with immune cells infiltrating (**Figure 5D**). The desert phenotype in IMvigor210 cohort was identified by histologically CD8+ T cell, and demonstrated a low response to atezolizumab. However, CD8+ T cell only explained 4% variance of response to atezolizumab (27). In lung adenocarcinoma (49) and acute myeloid leukemia (50), DNA methylation regulators defined low-risk group was preferentially associated with TME and the sensitivity to immune response. The inter-connectedness of immune factors and the gaps in our understanding of the mechanisms of response to immune-oncology agents means that CD8+ T cell alone will be not sufficient to deliver precision medicine across all the indications for ICIs. In this study, DMscore could be an independent variable to predict the response of ICIs. In brief, the DMscore could be an important biomarker for evaluating methylation modification patterns and predicting patient response to anti-CTLA-4/PD-1 immunotherapy.

Even though we analyzed multiple datasets, this study has a few limitations. First, we included DNA 5-mc methylation-related regulators in this study; future research could integrate additional m6A regulators to further advance the understanding of epigenetic regulation in HCC. Second, there is no accessible transcriptome or clinical data for ICI therapy patients with HCC. The predictive value of the DMscore for immunotherapy efficacy in HCC needs to be validated in further studies.

## Conclusions

In summary, this work investigated the comprehensive regulatory mechanisms of DNA 5-mc methylation modification in the HCC microenvironment and constructed a comprehensive scoring system for individual DNA methylation modification patterns. The DMscore serves as a valuable tool for predicting immune infiltration within the TME and refining the accuracy of immunotherapy prognosis.

## Data availability statement

The datasets presented in this study can be found in online repositories. The names of the repository/repositories and accession number(s) can be found below: NCBI SRA database, accession number: PRJNA762641.

## Ethics statement

The study received approval from the Research Ethics Committee at the First Affiliated Hospital of Zhejiang University, and written informed consent was acquired from all participating patients.

## Author contributions

JZ: Data curation, Formal Analysis, Funding acquisition, Writing – original draft. ZL: Methodology, Resources, Writing – original draft. KY: Resources, Validation, Writing – original draft. SS: Data curation, Methodology, Resources, Writing – original draft. JP: Conceptualization, Funding acquisition, Investigation, Supervision, Writing – review & editing.
