# Supplementary material for: DNA methylation regulator-based molecular subtyping and tumor microenvironment characterization in hepatocellular carcinoma
Source: Front Immunol. 2024 Apr 26;15:1333923. doi: 10.3389/fimmu.2024.1333923 (PMC11082416; doi:10.3389/fimmu.2024.1333923)
Supplement: Supplementary file 1 [file DataSheet_1.pdf]

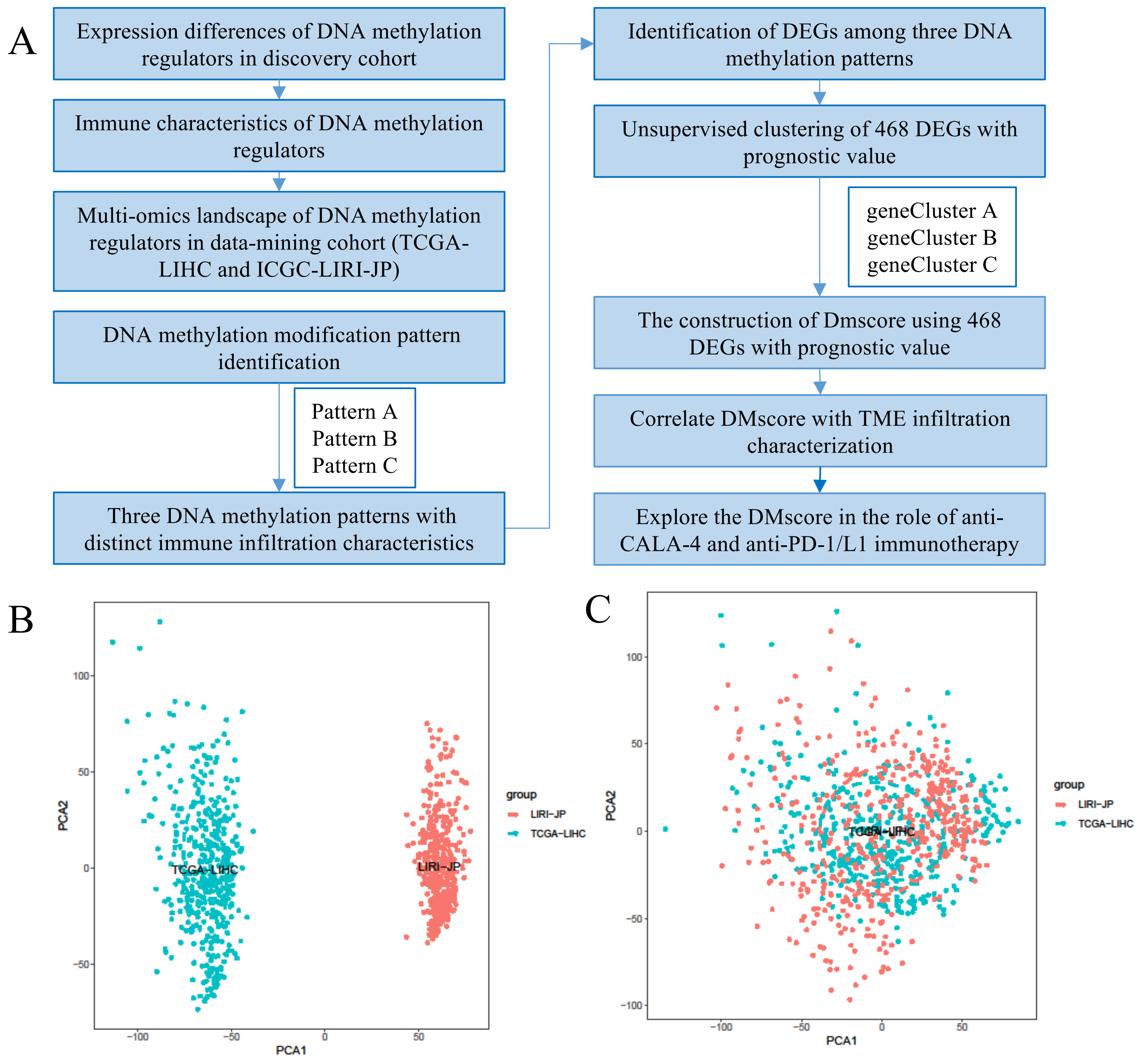

Fig. S1 Overview of study design and batch correction for data-mining cohort. (A) The workflow of our study design and analytical pipeline. (B) Principal component analysis (PCA) of mRNA expression data of sample from two HCC-cohorts before batch correction. (C) Principal component analysis (PCA) of mRNA expression data of sample from two HCC-cohorts after batch correction.

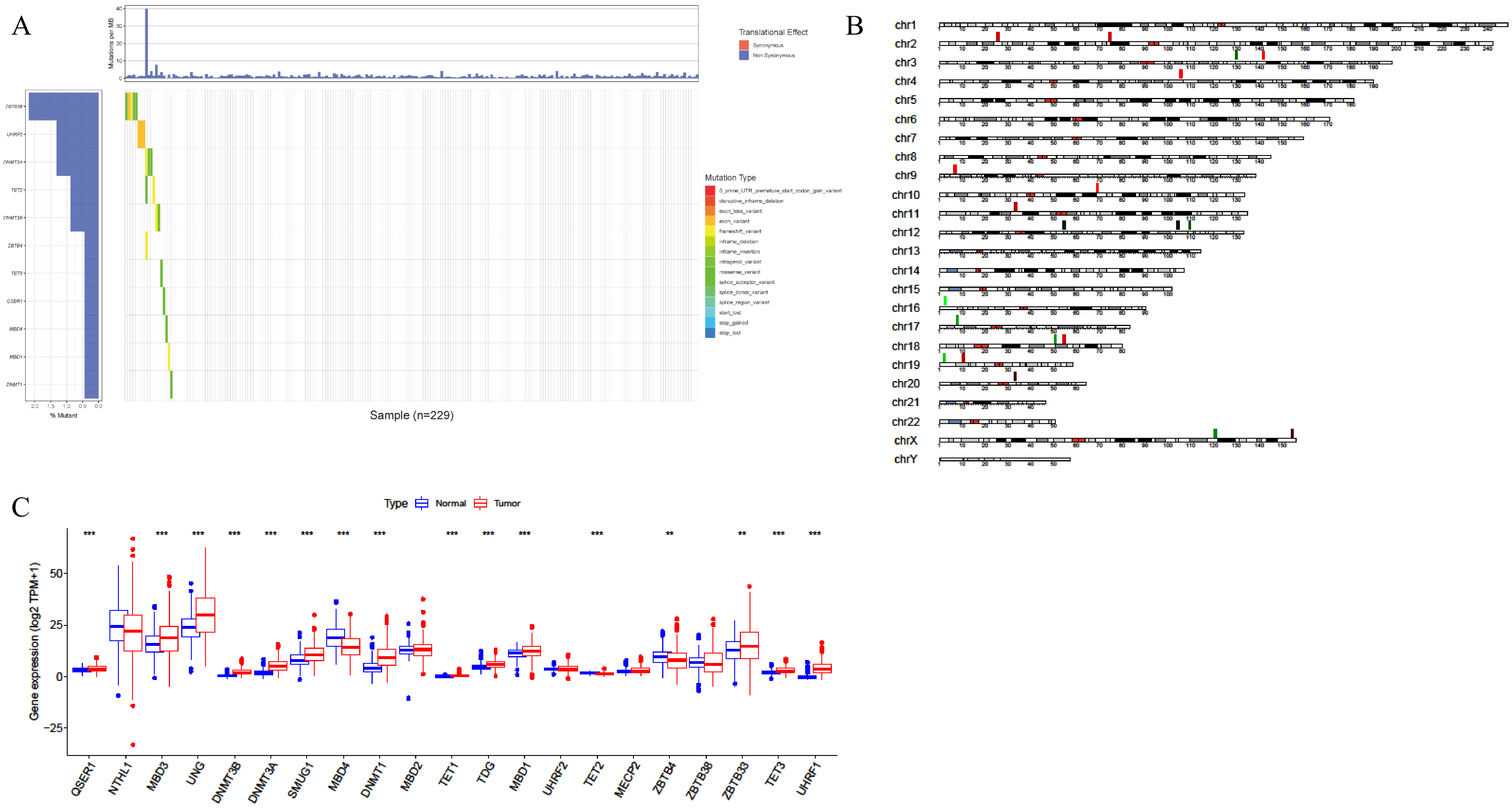

Fig. S2 Genetic mutation and expression of DNA methylation regulators in LIRI-JP cohort. (A) The mutation frequency of regulators in LIRI-JP cohort. Each column of the figure represents TMB. The number on the left shows the mutation frequency. (B) The location of the regulators on chromosomes. (C) Differences in gene expression levels of the regulators between normal and tumor patients in LIRI-JP cohorts. *p < 0.05, **p < 0.01, ***p < 0.001.

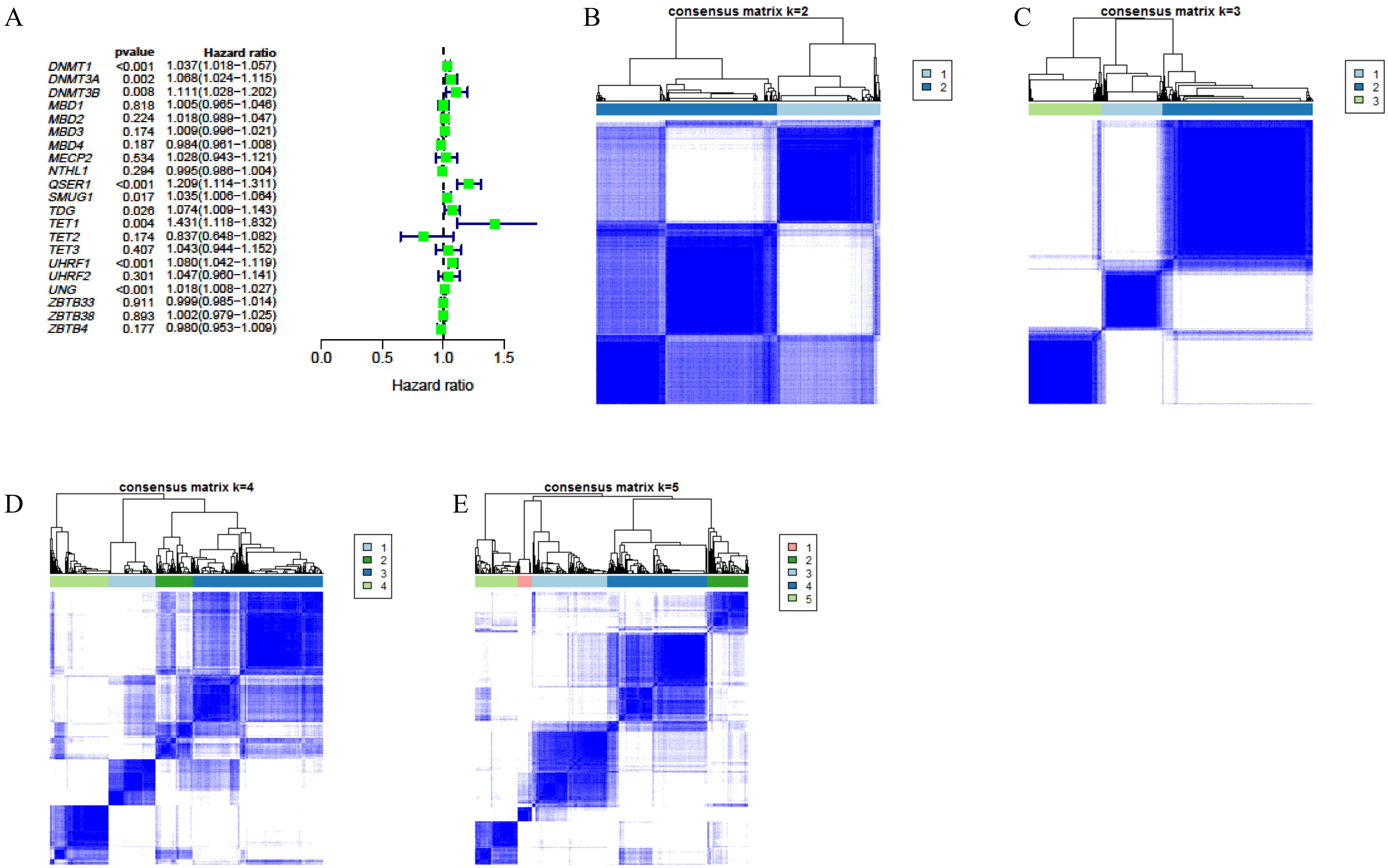

Fig. S3 Prognostic value and unsupervised clustering of 21 DNA methylation regulators. (A) The forest plot of the univariate Cox regression model depicting the prognostic value of 21 DNA methylation modifiers in the data-mining cohort. (B-E) The consensus clustering matrix (CM) plots of 21 DNA methylation regulator in the data-mining cohort (N = 608) for $k = 2$ to $k = 5$.

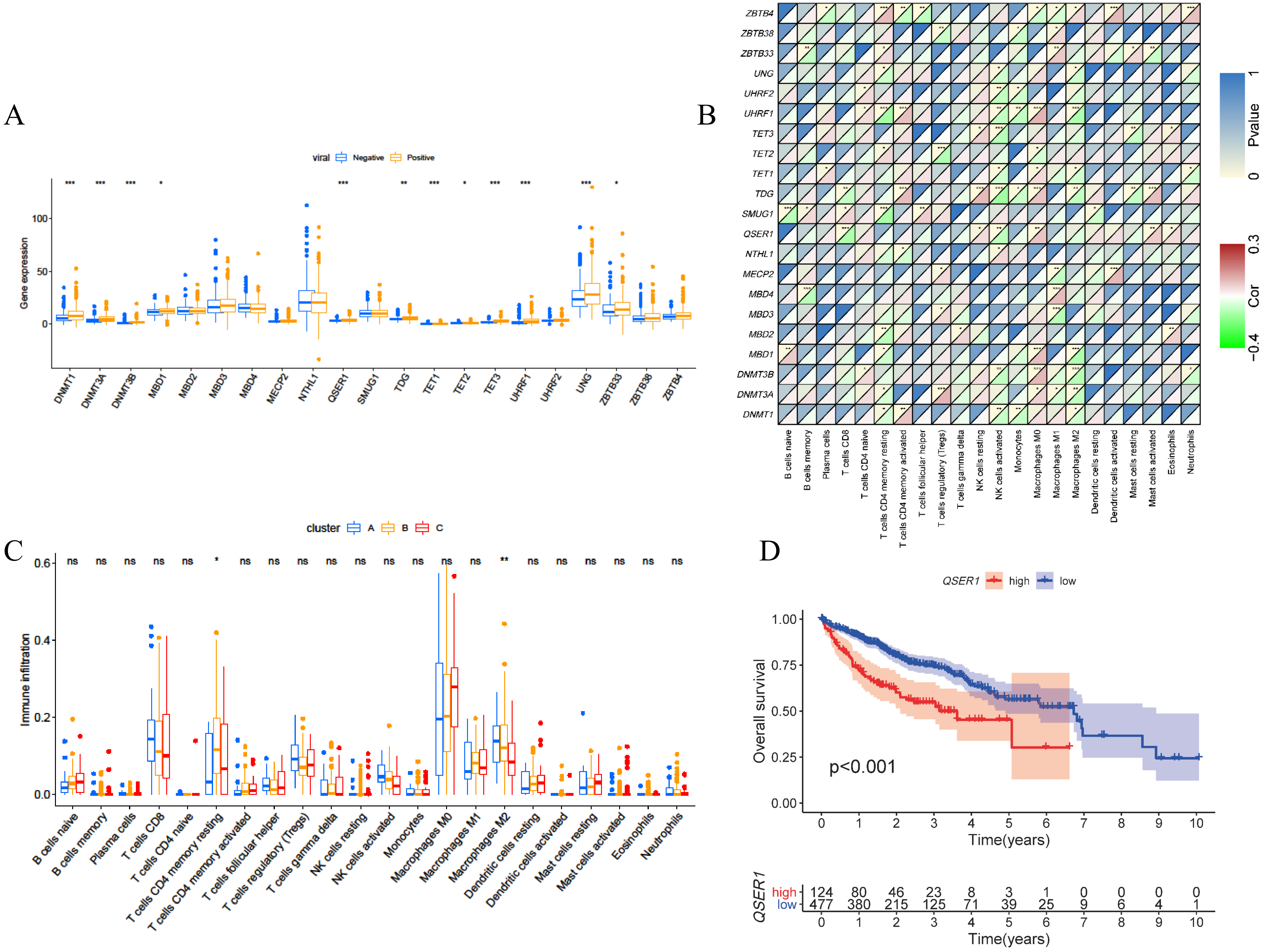

Fig. S4 DNA methylation regulators in HCC. (A) Difference in the expression of 21 DNA methylation regulators between HBV/HCV-positive and HBV/HCV-negative groups. The asterisks represented the statistical P value (*P < 0.05, **P < 0.01, ***P < 0.001). (B) Correlation heatmap between 20 DNA methylation regulators and immune cells in the data-mining cohort by CIBERSORT. (C) The difference of immune infiltration among DNA methylation patterns by CIBERSORT. The asterisks represented the statistical P value (*P < 0.05, **P < 0.01). (D) Overall survival analysis of high- (n = 124) and low- (n = 477) *QSER1* expression groups in the data-mining cohort. Log-rank test, p < 0.001.

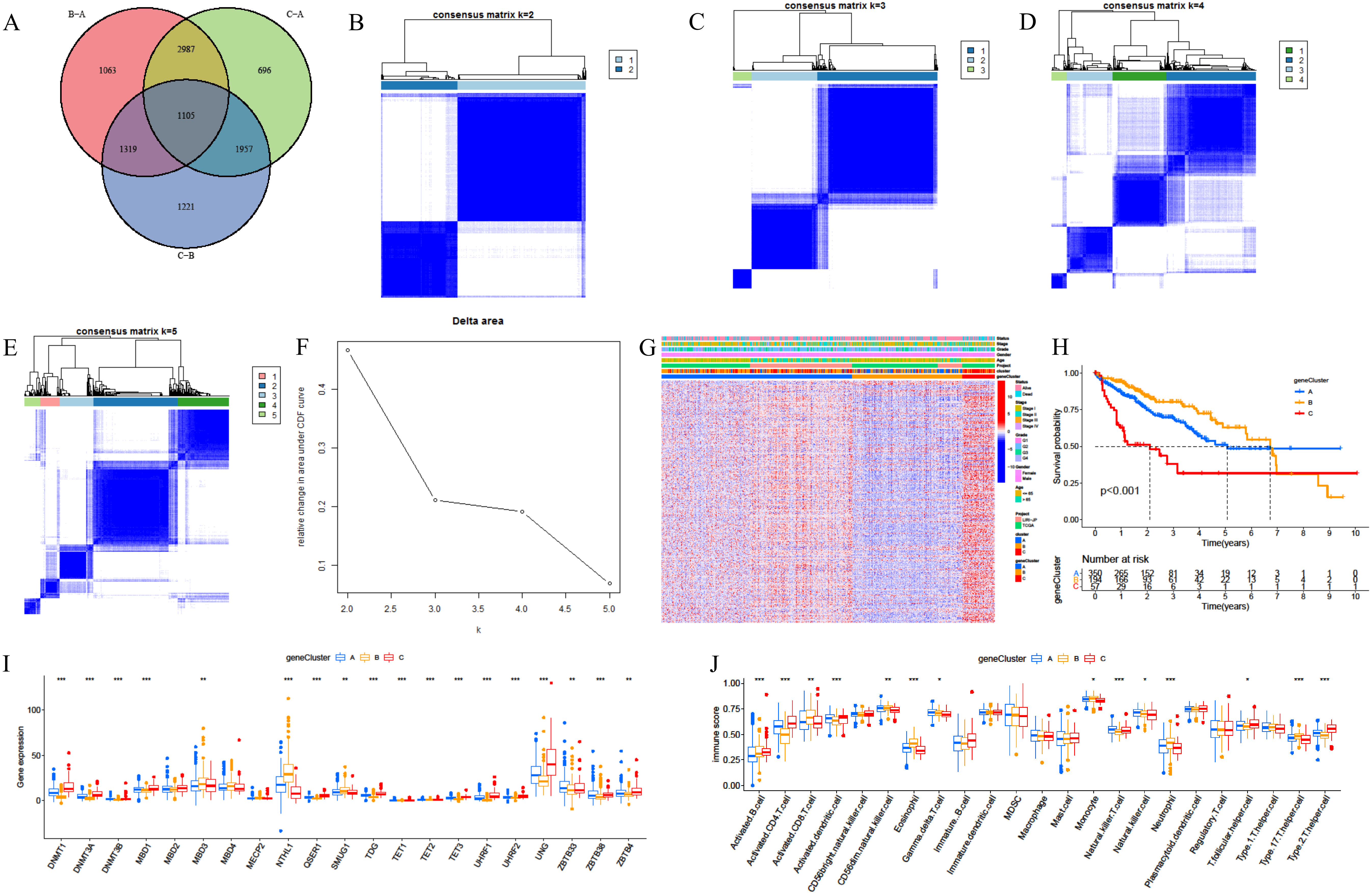

Fig. S5 Consensus clustering of DNA methylation signature genes. (A) 1105 DNA methylation phenotype-related genes shown in venn diagram. (B-E) The consensus clustering matrix (CM) plots of 468 DEGs with prognostic value in the data-mining cohort (N = 608) for $k = 2$ to $k = 5$. (F) Delta area curves for consensus clustering indicating the relative change in area under the cumulative distribution function (CDF) curve for each category number k compared to $k = 1$. The horizontal axis represents the category number k and the vertical axis represents the relative change in area under CDF curve. (G) The heatmap of 468 DEGs with prognostic value in three geneClusters in 608 data-mining cohort samples. (H) Kaplan-Meier plotter estimated the overall-survival outcome of the different geneClusters. Log-rank test, $P < 0.001$. (I) The expression level of 21 regulators in distinct gene clusters. The asterisks represented as the statistically significant p-values: $*P < 0.05$, $**P < 0.01$, $***P < 0.001$. (J) The immune cell infiltration in three geneClusters using the ssGSEA algorithm. The asterisks represented as the statistically significant p-values: $*P < 0.05$, $**P < 0.01$, $***P < 0.001$.

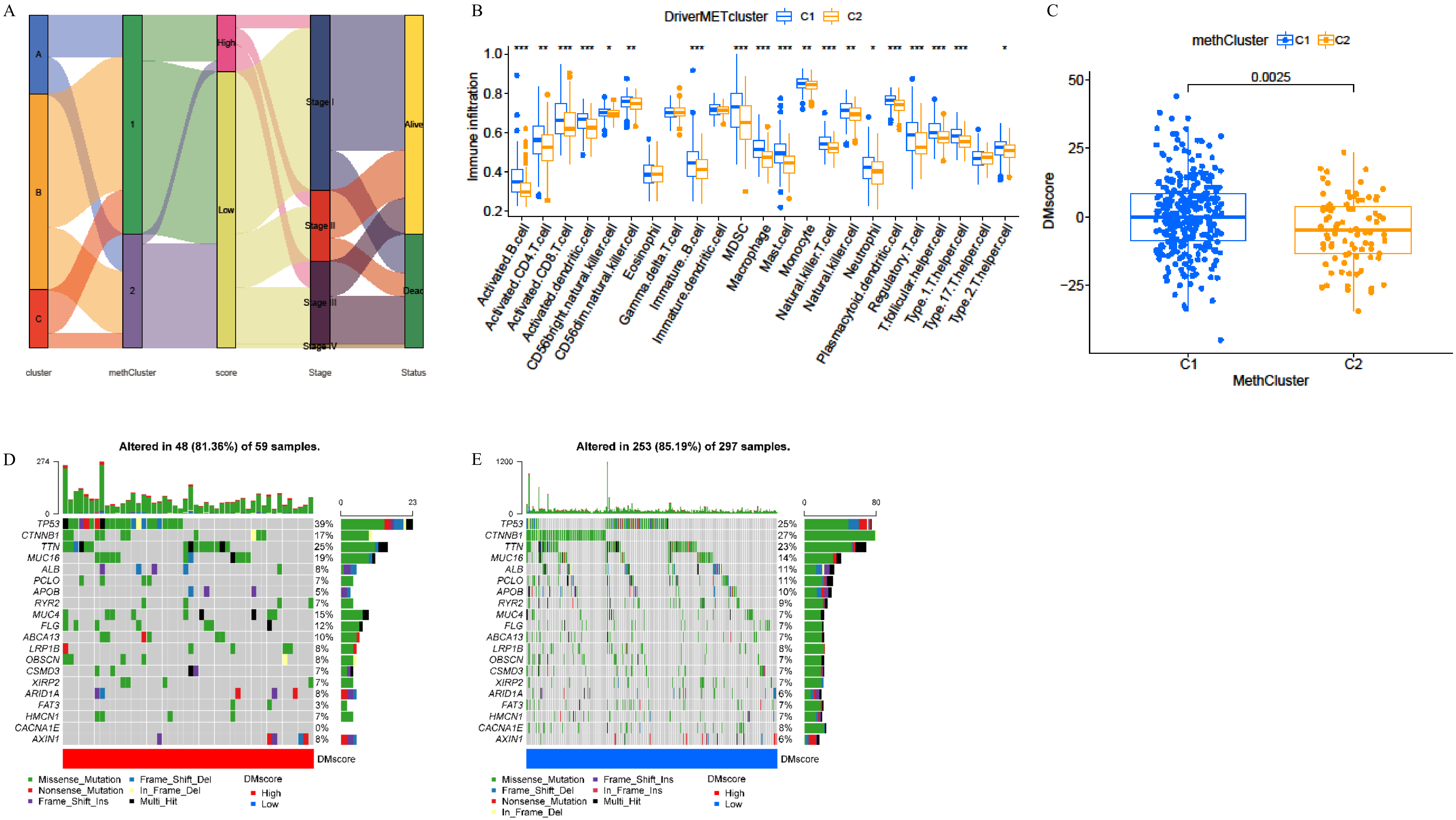

Fig. S6 Different immune characteristics of the MethClusters in TCGA-LIHC cohort. (A) Alluvial diagram showing the changes of methylation clusters, methCluster, DMscore, stage and living state. (B) The immune cell infiltration in three methClusters using the ssGSEA algorithm. The asterisks represented as the statistically significant p-values: *P < 0.05, **P < 0.01, ***P < 0.001. (C) Difference of DMscore between two methClusters. Wilcoxon test, P = 0.0025. (D, E) The landscape of tumor somatic mutation in TCGA-LIHC cohort evaluated between high (D) and low (E) DMscore. Each column represented individual patients. The upper barplot showed TMB, The number on the right indicated the mutation frequency in each gene. The right barplot showed the proportion of each variant type.

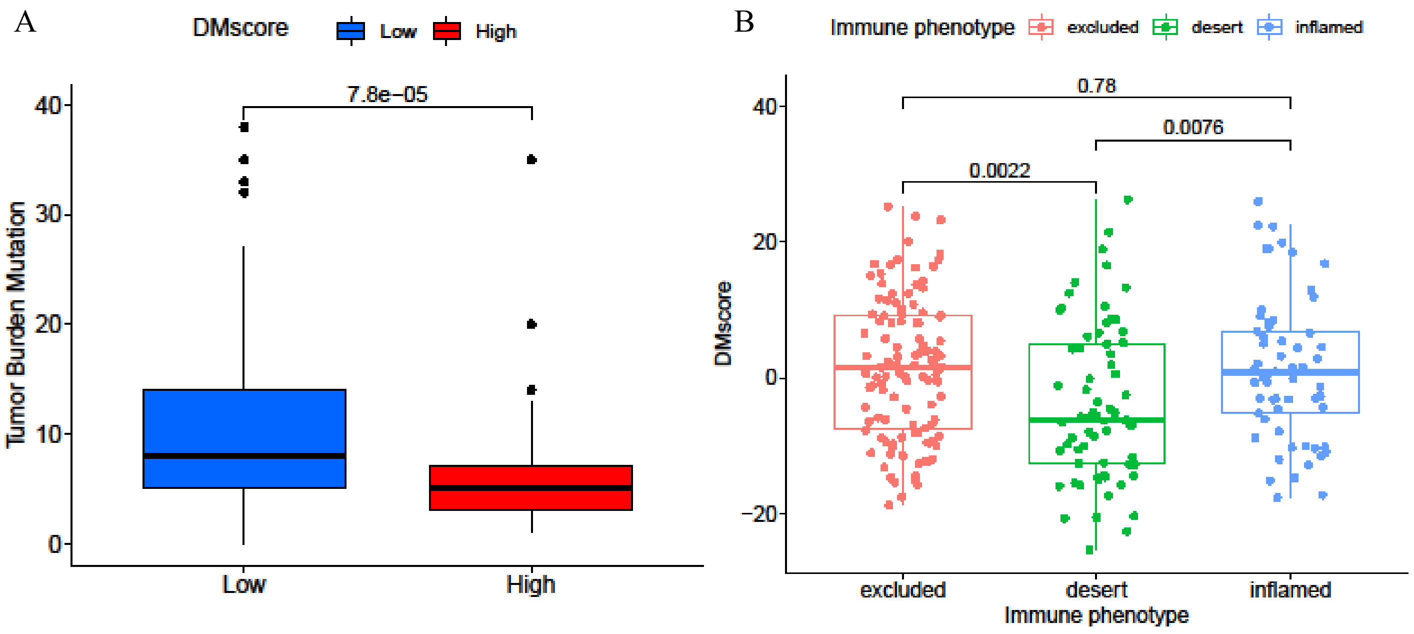

Fig. S7 The role of DMscore in anti-PD-L1 therapy (IMvigor210) cohort. (A) Boxplot of tumor mutation burden (TMB) for high- and low- DMscore groups. (B) Differences in DMscore among distinct tumor immune phenotypes in IMvigor210 cohort.

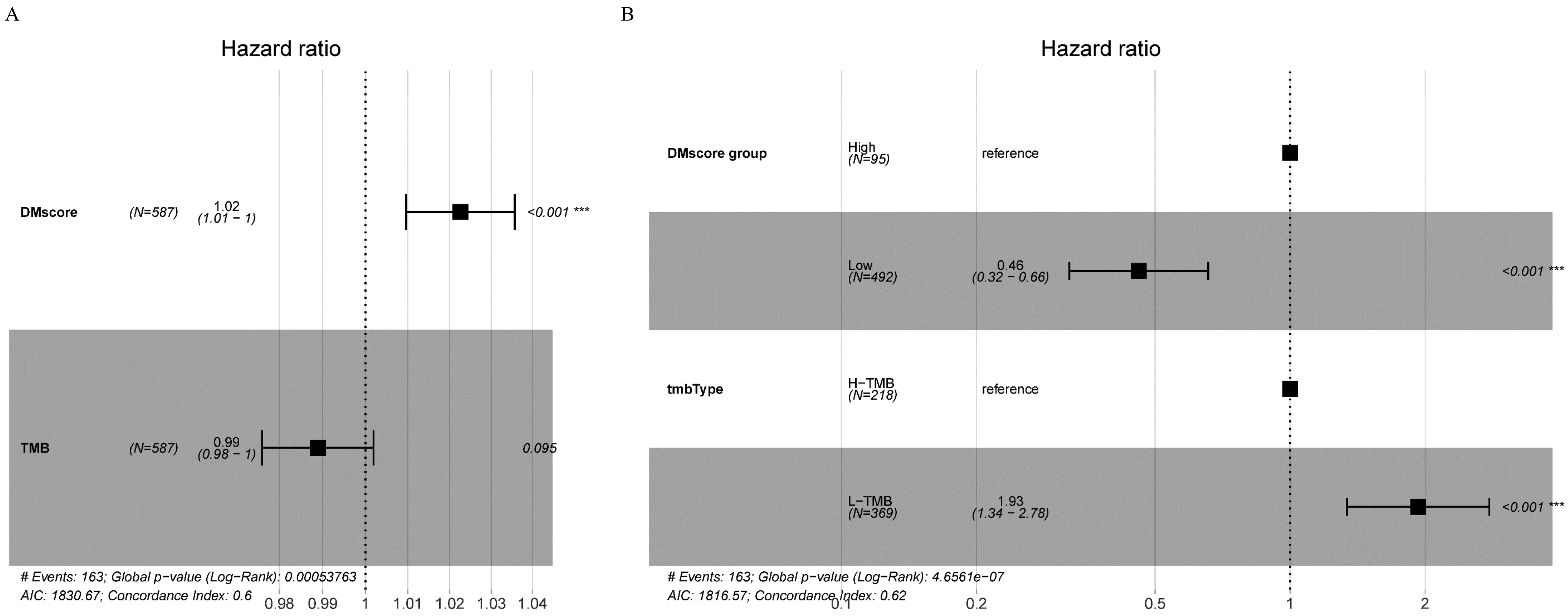

Fig. S8 The multiple cox regression analysis of DMscore and of tumor mutation burden (TMB) in anti-PD-L1 therapy (IMvigor210) cohort. (A) The multiple cox regression analysis of DMscore value and TMB number. (B) The multiple cox regression analysis of DMscore group and TMB type.
